# Colistin use and outcomes in Kuwait public hospitals

**DOI:** 10.3389/fdsfr.2026.1808461

**Published:** 2026-05-08

**Authors:** Sarah S. Alghanem, Wadha A. Alfouzan, Moetaza M. Soliman, Ebtehal Alroomi, Salma Alajmi, Tarek Mahmoud, Jude Yagan

**Affiliations:** 1 Department of Pharmacy Practice, College of Pharmacy, Kuwait University, Kuwait, Kuwait; 2 Department of Microbiology College of Medicine, Kuwait University, Safat, Kuwait; 3 Department of Laboratory Medicine, Farwaniya Hospital, Ministry of Health, Sulaibikhat, Kuwait; 4 Department of Clinical Pharmacy and Pharmacy Practice, Faculty of Pharmacy, Mansoura University, Mansoura, Egypt; 5 Department of Laboratory Medicine, Al-Jahra Hospital, Kuwait Ministry of Health, Sulaibikhat, Kuwait; 6 Department of Laboratory Medicine, Al-Adan Hospital, Kuwait Ministry of Health, Sulaibikhat, Kuwait; 7 Hamed Al-Essa Organ Transplant Centre, Kuwait Ministry of Health, Sulaibikhat, Kuwait

**Keywords:** clincal outcomes, gram-negative bacteria, loading dose, MDR infections, nephrotoxicity

## Abstract

**Background:**

In Kuwait, colistin use has increased owing to an increased incidence of multidrug resistant infections. However, the optimal dosing regimen and its effectiveness and safety are unclear. We aimed to describe colistin use in Kuwait and evaluate the associated clinical outcomes and safety profiles.

**Methods:**

This multicentre, retrospective, observational study used data collected from five hospitals. Study outcomes included cure rates, incidence of nephrotoxicity, and mortality. Logistic regression models were used to study the effect of colistin dosing patterns (loading dose, daily maintenance dose, and duration of therapy) on study outcomes. The models were adjusted for age, sex, and hospital unit (ICU/non-ICU), baseline creatinine clearance, being on dialysis, pathogen type, receiving concomitant antibiotics, and site of infection.

**Results:**

Of 205 patients treated with colistin, 117 were treated in intensive care units (ICUs) and 88 were treated in other units. Of the 205 patients, 145 (70.7%) achieved clinical cure. The cure rate was lower in ICU patients than in non-ICU patients. Fifty patients experienced nephrotoxicity; 47 died, and 11 experienced neurotoxicity. The incidence of nephrotoxicity did not differ significantly by hospital unit, whereas the case fatality rate was significantly higher and the incidence of neurotoxicity was significantly lower in ICU patients. Administration of a loading dose was not associated with the cure rate, incidence of nephrotoxicity, or case fatality rate. Higher maintenance doses were associated with higher cure rates, (odds ratio [OR], 2.77; 95% confidence interval [CI], 1.04–7.37) and lower fatality rates (OR, 0.29; 95% CI, 0.09–0.95), but more likely to develop nephrotoxicity (OR, 2.60; 95% CI, 1.08–6.23).

**Conclusion:**

Administration of high maintenance doses of colistin was associated with higher cure rates but was also associated with a higher incidence of nephrotoxicity.

## 1 Introduction

Colistin is the active form of colistimethate sodium, which was developed in the late 1940s (Kwa et al., 2007). However, its use is limited owing to nephrotoxicity and the availability of safe alternatives. In recent years, the incidence of multidrug-resistant (MDR) infections has increased with limited available treatment options. In Kuwait, the incidence of MDR infections is increasing, with 37.2% of *Acinetobacter* cultures being resistant to meropenem and 12% being resistant to colistin (Al-Sweih et al., 2011). Resistance rate among MDR *Pseudomonas aeruginosa* isolates to meropenem was high (≥87.5%) and low resistance rates to colistin (2.1%) were observed from Kuwait (Alatoom et al., 2024), while the reported *Klebsiella pneumoniae* resistant to colistin was 8% (Shahimi et al., 2025). Therefore, colistin has re-emerged as a drug of last resort for treating patients with MDR infections.

A systematic review showed that in Middle Eastern countries, the median MDR prevalence was 74% for carbapenem-resistant *Acinetobacter* spp., 8% for *Escherichia coli*, and 15% for *K. pneumoniae* (Truppa and Abo-Shehada, 2020). Dosage regimen recommended by the manufacturer had reported a high rates of treatment failure (Dijkmans et al., 2015; Antoniadou et al., 2007; Ko et al., 2007). Studies have shown that older pharmacokinetic evaluations of colistin may be inaccurate because of the failure of previous assays to differentiate between colistimethate sodium (the prodrug) and colistin (the active drug) (Dijkmans et al., 2015). Recently, colistin has been subjected to rigorous studies to optimise dosing regimens and evaluate its efficacy, in accordance with regulatory agency requirements for approving new drugs (Mohamed et al., 2014; Mohamed et al., 2012; Nation et al., 2017; Plachouras et al., 2009).

Modelling and simulation studies have suggested that high loading and maintenance doses of colistin are more effective for critically ill patients (Mohamed et al., 2014; Mohamed et al., 2012; Plachouras et al., 2009). In December 2014, the European Medicines Agency approved changes in the colistin dosage regimen, with the administration of a loading dose and an increase in the maintenance dose (European Medicines Agency, 2014). Previous research (Wertheim et al., 2013) reported major variability in the dosing regimens used in different countries, with only 21% of the patients studied receiving loading doses for colistin. In Kuwait, some institutions rely on the European Medicines Agency dosing recommendations (European Medicines Agency, 2014), whereas others rely on the pharmacokinetic working group dosing recommendations (Nation et al., 2017). The international consensus guidelines for the optimal use of polymyxins were published in 2019 to provide clinical guidance for the use of these agents (Tsu et al., 2019). However, the efficacy and safety of the recommended dosing regimens have not been confirmed, and the optimal colistin dosing regimen is unclear. In an age of increased vigilance over antibiotic stewardship in the face of increasing MDR pathogens globally and nationally, investigating the current colistin-use practices in Kuwait is essential to maximise the dosing effectiveness, minimise toxicity, and establish consensus among health institutions. Therefore, we aimed to assess the patterns of colistin use in Kuwait among critically ill and non-critically ill patients and evaluate the associated clinical outcomes and safety profiles.

## 2 Methods

### 2.1 Study design and population

This was a multicentre, retrospective, observational study. Data were collected from four secondary hospitals in Kuwait and one tertiary hospital specialising in organ transplantation, primarily kidney transplantation. The study included all patients hospitalised between September 2017 and April 2019 who received colistin during hospitalisation. Ethical approval was obtained from the Health Science Center and Ministry of Health Ethical Committees (MoH/REC/625/2017). The ethics committee review board waived the requirement of written informed consent for participation from the participants or the participants’ legal guardians/next of kin because of the retrospective nature of the study.

Patients aged ≥18 years, with documentation of infection with an MDR gram-negative pathogen, who received colistin therapy for at least 3 days were eligible for inclusion in the analysis. Both intensive care unit (ICU) and non-ICU patients were included. Patients who were neutropenic; with sterile culture or a culture with non-MDR gram-negative pathogens; and those with missing data on age, sex, or colistin dosing were excluded.

All data were collected from patients’ medical files (either paper or electronic records). The data included baseline patient characteristics such as age, sex, and comorbidities. Patients’ clinical and laboratory data included temperature, white blood cell count, platelet count, and procalcitonin levels. Data were also collected on the dosing regimen of colistin, including the loading dose, maintenance dose, and duration of colistin therapy. Data on the culture results and the infection site were collected at baseline and at the end of colistin therapy. The method used to determine colistin susceptibility is the manual broth micro-dilution (mBMD) and the automated system (Vitek 2, Biomeriux, France), he and the Clinical and Laboratory Standards Institute is used for interpretation criteria.

### 2.2 Clinical outcomes

The primary clinical outcomes included clinical cure and all-cause mortality, and the secondary outcomes included the incidence of nephrotoxicity and neurotoxicity. Clinical cure was defined as the patient (i) being alive, (ii) having a body temperature of <38 °C and no recurrence for 48 h, and (iii) an improvement in the signs and symptoms at the infection site (as reported by the clinician in the medical file), (iv) a white blood cell count of <12 × 109/L, and (v) with or without a negative culture documented at the end of colistin therapy (Dalfino et al., 2012; Karnik et al., 2013; Vicari et al., 2013). Mortality was defined as death d during treatment. Nephrotoxicity was defined as the development of acute kidney injury (AKI), which was defined according to the Kidney Disease: Improving Global Outcomes criteria as an increase in serum creatinine ≥1.5 times the baseline in 7 days or a urine volume of <0.5 mL/kg/h for >6 h (Khwaja, 2012). Neurotoxicity was defined as documentation of peripheral and orofacial paraesthesia, visual disturbances, vertigo, mental confusion, ataxia, seizures, or other manifestations (e.g., neuromuscular blockade presenting as a myasthenia-like syndrome or respiratory muscle paralysis producing apnoea).

### 2.3 Statistical analysis

Descriptive statistics were used to describe the baseline characteristics and patient outcomes. The patients were stratified according to whether they were admitted to an ICU or another hospital unit. The Mann–Whitney U test was used to compare the groups if the data were non-normally distributed. The proportions of patients who achieved clinical cure, developed AKI, or died were reported as frequencies and percentages. The Chi-square test was used to compare proportions between groups. Logistic regression models were used to study the effect of colistin dosing patterns (loading dose, daily maintenance dose, and duration of therapy) on clinical outcomes (clinical cure, incidence of AKI, and mortality). The models were adjusted for age, sex, hospital unit (ICU/non-ICU), baseline creatinine clearance, being on dialysis, pathogen type, receiving concomitant antibiotics, and site of infection. The results of the regression models were reported as odds ratios (ORs) with 95% confidence intervals (CIs). Statistical significance was set at P < 0.05. All analyses were performed using Stata 10.1 software (Stata Corp.; College Station, TX, United States).

## 3 Results

### 3.1 Baseline patient characteristics

This study included 205 patients from five hospitals. Out of 245 patients whose files were reviewed, 40 were excluded because of incomplete information. Of the 245 patients, 117 (57.1%) were treated in ICUs, and 88 (42.9%) patients were treated in other units. The baseline patient characteristics are summarised according to hospital unit (ICU/non-ICU) in Table 1. The median (interquartile range [IQR]) age of the patients was 60.0 (43.0–72.0) years, and 112 (54.6%) patients were men. Most common comorbidities included hypertension (66.3%), diabetes mellitus (52.2%), dyslipidaemia (22.4%), cardiovascular disease (34.6%), and chronic kidney disease (18.5%). Compared with non-ICU patients, a higher proportion of ICU patients had chronic kidney disease, and a lower proportion had hypertension and dyslipidaemia. Additionally, compared with non-ICU patients, a higher proportion of ICU patients were on dialysis (80.6% vs. 19.4%, *P* = 0.002), mainly continuous renal replacement therapy.

**TABLE 1:** Baseline demographics of the patients.

​	ICU patients (n = 117)		Non-ICU patients (n = 88)		Total cohort (n = 205)		P value*
Age in years, median (IQR)	60.0	(43–72)	60.0	(45–72)	60.0	(43–72)	0.49
Gender, n (%)							
Male	66	(56.4)	46	(52.3)	112	(54.6)	0.56
Female	51	(43.6)	42	(47.7)	93	(45.4)	
Weight in kg, median (IQR)	80	(68–88.4)	78	(69–87.5)	80	(68–88.4)	0.53
Height in cm, median (IQR)	160	(156–170)	160.5	(153–166)	160	(154–168)	0.27
Comorbidities, n (%)							
Hypertension	70	(59.8)	66	(75.0)	136	(66.3)	0.02
DM	56	(47.9)	51	(58.0)	107	(52.2)	0.15
Dyslipidemia	13	(11.1)	33	(37.5)	46	(22.4)	<0.001
CVD	38	(32.5)	33	(37.5)	71	(34.6)	0.18
CKD	30	(25.6)	8	(9.1)	38	(18.5)	0.01
Site of infection, n (%)							
Blood	23	(19.7)	6	(6.8)	29	(14.1)	<0.001
Chest	53	(45.3)	16	(18.2)	69	(33.7)	
Skin and soft tissue	6	(5.1)	14	(15.9)	20	(9.8)	
Urine	7	(6.0)	33	(37.5)	40	(19.5)	
Multiple site infection	28	(23.9)	19	(21.6)	47	(22.9)	
Concomitant antibiotic therapy, n (%)							
Carbapenems	45	(38.5)	26	(29.5)	71	(34.6)	0.18
Piperacillin/tazobactam	31	(26.5)	13	(14.8)	44	(21.5)	0.04
Quinolones	15	(12.8)	8	(9.1)	23	(11.2)	0.40
Tigecycline	6	(5.1)	6	(6.8)	12	(5.9)	0.61
Monotherapy	40	(34.2)	41	(46.6)	81	(39.5)	0.07
Clinical and laboratory data, median (IQR)							
Temperature in C°	37	(37–38)	37	(36.7–38)	37	(36.8–38)	0.27
WBC in x 10^9^/L	13	(9.2–17.6)	9.0	(7.5–12.9)	12	(8.2–16.3)	<0.0001
Platelet count in × 10^9^/L	204	(98–309)	276.5	(182–368)	231	(138–340)	0.001
Procalcitonin in ng/mL	1.6	(0.5–9)	0.4	(0.1–1.8)	0.9	(0.2–5.4)	<0.0001
SCr in µmol/L	145	(63–280)	106	(68.5–157)	124	(67.5–230)	0.08
BUN in mmol/L	13.4	(8.2–26.1)	8.2	(5.4–13.3)	10.7	(6.5–19.9)	<0.0001
Renal replacement therapy, n (%)							
On dialysis	29	(80.6)	7	(19.4)	36	(17.6)	0.002
Type of dialysis, n (%)							
Continuous renal replacement therapy	25	(86.2)	4	(57.1)	29	(80.6)	0.14
Hemodialysis	2	(6.9)	1	(14.3)	3	(8.3)	
Peritoneal dialysis	1	(3.4)	0	(0.0)	1	(2.8)	​
Missing	1	(3.4)	2	(28.6)	3	(8.3)	

BUN, blood urea nitrogen; CKD, chronic kidney disease; CVD, cardiovascular disease; DM, diabetes mellitus; ICU, intensive care unit; IQR, interquartile range; SCr, serum creatinine; WBC, white blood cell.

* Mann-Whitney U test was used for continuous data and chi-square test for proportions.

Infection sites included the chest (33.7%), urinary tract (19.5%), blood (14.1%), and skin and soft tissue (9.8%). Of the 205 patients, 47 (22.9%) had infections at two or more sites. Chest infection was most common in ICU patients (45.3%), whereas urinary tract infection was most common in non-ICU patients (37.5%). Compared with non-ICU patients, ICU patients had higher bilirubin, procalcitonin, and blood urea nitrogen levels, higher white blood cell counts, and lower platelet counts. Colistin was used as monotherapy in 81 (39.5%) patients, and administered in combination with other antibiotics such as carbapenems, piperacillin/tazobactam, quinolone, or tigecycline in the rest of the patients (Table 1).

The most frequently detected pathogen was *A. baumannii*, which was detected in 123 patients (60%). Other pathogens included *P. aeruginosa* (62 patients, 30.2%), *K. pneumoniae* (44 patients, 21.5%), *E. coli* (12 patients, 5.9%), *Enterobacter cloacae* (3 patients, 1.5%), and *Stenotrophomonas maltophilia* (1 patient, 0.5%). All cultures were susceptible to colistin. *Acinetobacter baumannii* was more common among ICU patients than non-ICU patients, whereas *P. aeruginosa* and *Acinetobacter baumannii* were more common among non-ICU patients (Supplementary Table S1).

### 3.2 Colistin dosing regimens

**FIGURE 1:**
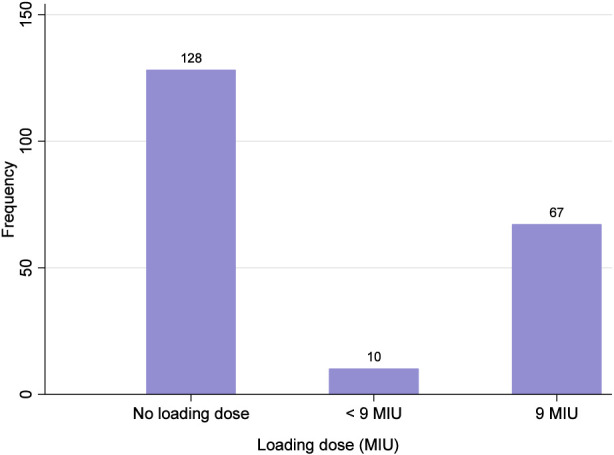
The administered loading dose of colistin.

Of the patients, 128 (62.4%) did not receive a loading dose of colistin. This proportion was significantly higher in non-ICU patients than in ICU patients (85.2% vs. 45.3%; P < 0.001; Table 2). Among the patients who received a loading dose of colistin, the majority (87.0%) received 9 million international units (MIU) (Figure 1).

**TABLE 2:** Pattern of colistin dosing.

​	ICU patients (n = 117)		Non-ICU patients (n = 88)		Total cohort (n = 205)		P value*
Administration of a loading dose, n (%)							
No loading dose	53	(45.3)	75	(85.2)	128	(62.4)	<0.001
Loading dose (6–9 MIU)	64	(54.7)	13	(14.8)	77	(37.6)	
Administered loading dose according to the protocol used, n (%) (n = 77)							
Equal to recommended dose	45	(70.3)	7	(53.8)	52	(67.5)	0.50
Lower than recommended dose	4	(6.3)	1	(7.7)	5	(6.5)	
Higher than recommended dose	15	(23.4)	5	(38.5)	20	(26.0)	
Time taken to start maintenance dose after the loading dose, n (%) (n = 77)							
Immediately after loading dose	26	(40.6)	6	(46.2)	32	(41.6)	0.14
After 8 h	24	(37.5)	1	(7.7)	25	(32.5)	
After 12 h	12	(18.8)	5	(38.5)	17	(22.1)	
After 24 h	2	(3.1)	1	(7.7)	3	(3.9)	
Frequency of maintenance dose (n times per day), n (%)							
Every 8 h	59	(50.4)	37	(42.0)	96	(46.8)	0.33
Every 12 h	54	(46.2)	45	(51.1)	99	(48.3)	
Every 24 h	4	(3.4)	6	(6.8)	10	(4.9)	
Colistin daily maintenance dose in MIU, median (IQR)	6	(3–9)	5	(3–6)	5	(3–9)	0.002
Duration of therapy in days, median (IQR)	12	(8–15)	12	(10–14)	12	(8–14)	0.98
Colistin cumulative dose, in MIU, median (IQR)	55	(36–99)	47.8	(33–70)	54	(36–90)	0.04

ICU, intensive care unit; IQR, interquartile range; MIU, million international unit.

* Mann-Whitney U test was used for continuous data and chi-square test for proportions.

The administered maintenance dose regimens for colistin ranged from 0.5 to 4.5 MIU every 8, 12, or 24 h. The most frequent daily maintenance dose was 9 MIU, administered to 57 (36.6%) patients either as 3 MIU every 8 h (39 patients, 19.0%) or as 4.5 MIU every 12 h (18 patients, 8.8%). The median [IQR] daily maintenance dose was significantly higher in ICU patients than in non-ICU patients (6.0 [3.0–9.0] MIU *versus* 5.0 [3.0–6.0] MIU; P = 0.002) (Table 2). The median [IQR] daily maintenance dose was lower in patients who were on dialysis than in those who were not (3.0 [2.3–4.5] MIU *versus* 6.0 [3.0–9.0] MIU; P < 0.001). The median (IQR) duration of colistin therapy was 12.0 (8.0–14.0) days, with no difference according to hospital unit (Table 2).

### 3.3 Outcomes of colistin therapy

A total of 145 (70.7%) patients achieved clinical cure. ICU patients showed cure rates lower than those of non-ICU patients (59.8% vs. 85.2%, respectively; P < 0.001; Table 3). Fifty patients (24.4%) developed AKI, and 47 patients (22.9%) died. Deaths were more common with ICU patients than with non-ICU patients (33.3% vs. 9.1%, respectively; P < 0.001). Neurotoxicological symptoms were reported in 11 (5.4%) patients, including peripheral and orofacial paraesthesia (5 patients), mental confusion (2 patients), ataxia (3 patients), and other symptoms (1 patient). Neurological symptoms were more commonly documented in non-ICU patients than in ICU patients.

**TABLE 3:** Outcomes of colistin therapy.

​	ICU patients (n = 117)		Non-ICU patients (n = 88)		Total cohort (n = 205)		P value*
Clinical cure, n (%)	70	(59.8)	75	(85.2)	145	(70.7)	<0.001
AKI, n (%)	28	(23.9)	22	(25.0)	50	(24.4)	0.72
Deaths, n (%)	39	(33.3)	8	(9.1)	47	(22.9)	<0.001
Neurotoxicity, n (%)							
Peripheral and orofacial paresthesia	0	(0)	5	(5.7)	5	(2.4)	<0.001
Mental confusion	2	(1.7)	0	(0)	2	(1.0)	
Ataxia	1	(0.9)	2	(2.3)	3	(1.5)	
Other	0	(0)	1	(1.1)	1	(0.5)	
Hospital length of stay in days, median (IQR)	19.5	(10.5–32)	20.5	(10.5–38)	20.0	(10.5–34)	0.37

AKI, acute kidney injury; ICU, intensive care unit; IQR, interquartile range.

* Mann-Whitney U test was used for continuous data and chi-square test for proportions.

### 3.4 Factors associated with the clinical outcomes of colistin therapy

The results of the unadjusted logistic regression analysis are presented in Supplementary Table S2. Compared with colistin monotherapy, concomitant antibiotic therapy (carbapenem, piperacillin/tazobactam, quinolone, or tigecycline) did not improve clinical cure rate (OR, 0.88; 95% CI, 0.48–1.63; P = 0.68). The cure rates did not differ significantly according to whether patients were infected with *A. baumannii* (OR, 0.60; 95% CI, 0.32–1.14; P = 0.12), *P. aeruginosa* (OR, 1.44; 95% CI, 0.73–2.84; P = 0.29), or *Klebsiella pneumonia* (OR, 1.80; 95% CI, 0.81–4.03; P = 0.15).

Multivariable logistic regression analysis, adjusted for age, sex, hospital unit, baseline creatinine clearance, being on dialysis, pathogen type, receiving concomitant antibiotics, and site of infection, showed that receiving a loading dose of colistin did not affect the clinical cure rate, AKI incidence, or mortality (Table 4). Patients who received a daily maintenance dose of colistin ≥9 MIU had higher cure rates (OR, 2.77; 95% CI, 1.04–7.37; P = 0.04) and lower case fatality rates (OR, 0.29; 95% CI, 0.09–0.95; P = 0.04) than were those who received a daily maintenance dose <9 MIU; however, they were more likely to develop AKI (OR, 2.60; 95% CI, 1.08–6.23; P = 0.003). Patients who received colistin for a long duration were less likely to die (OR, 0.87; 95% CI, 0.80–0.95; P = 0.02) than were those who received colistin for a short duration (Table 4). Older age was a risk factor for poor cure rates, a high incidence of AKI, and mortality (Supplementary Table S3).

**TABLE 4:** Multivariable logistic regression of clinical outcomes*.

​	Clinical cure			AKI			Death		
	OR	95% CI	P value	OR	95% CI	P value	OR	95% CI	P value
No loading dose	References								
Receiving a loading dose	0.67	[0.29,1.57]	0.36	0.96	[0.41,2.24]	0.93	1.96	[0.75,5.13]	0.17
Daily maintenance dose <9 MIU	References								
Daily maintenance dose ≥9 MIU	2.77	[1.04,7.37]	0.04	2.60	[1.08,6.23]	0.03	0.29	[0.09,0.95]	0.04
Duration of therapy (days)	1.07	[1.00,1.14]	0.05	1.06	[0.99,1.12]	0.05	0.87	[0.80,0.95]	0.002

AKI, acute kidney injury; CI, confidence interval; ICU, intensive care unit; LD, loading dose; OR, odds ratio; MD, maintenance dose.

* The models were adjusted for age, sex, and hospital unit (ICU/non-ICU), baseline creatinine clearance, being on dialysis, pathogen type, receiving concomitant antibiotics, and site of infection.

## 4 Discussion

This study assessed real-life usage patterns and associated clinical outcomes of colistin use in Kuwait. The chest was the most common infection site, and *A. baumannii* was the main pathogen. Administration of a colistin loading dose was not associated with the clinical cure rate, incidence of nephrotoxicity, or case fatality rate. In contrast, administration of a high maintenance dose of colistin was associated with higher cure rates and survival rates but was also associated with a high incidence of nephrotoxicity. These results are consistent with those of a recent meta-analysis comprising eight studies and 1,115 patients, which concluded that the administration of a colistin loading dose in patients receiving high maintenance dose regimens was significantly associated with higher microbiological response rates but that this did not affect the clinical cure rate, case fatality rate, or risk of nephrotoxicity (Bellos et al., 2020). The findings of this study support the administration of a high maintenance dose of colistin, as recommended by the international consensus therapeutic guidelines (Tsu et al., 2019), but further research is required to evaluate the benefit of colistin loading doses, ideally by means of randomised controlled trials. Such studies should compare fixed and variable loading doses, combined with high maintenance dose regimens, as proposed by Gontijo and Cavalieri (Gontijo and Cavalieri, 2023). In addition, different colistin dosage regimens should be evaluated in both critically ill and non-critically ill patients who require colistin therapy.

The international consensus therapeutic guidelines recommend a loading dose of 9 MIU colistin to reach a plasma colistin concentration of 2 mg/L and administration of the next dose after 12–24 h (Tsu et al., 2019). However, a recent simulation study suggested that loading dose regimens, including the dose and waiting time before the maintenance dose, should be adjusted according to the patient’s renal function and weight (Gontijo and Cavalieri, 2023). The simulations showed that a loading dose of 9 MIU of colistin and a waiting time of 24 h to start the maintenance dose resulted in subtherapeutic concentrations in those with high creatinine clearance and supratherapeutic colistin concentrations in those with poor renal function. However, pharmacokinetic studies have suggested that the optimal time to initiate the maintenance dose is 24 h after the loading dose (Garonzik et al., 2011). This study did not assess the effect of timing of initiation of the maintenance dose following the administration of the loading dose on the risk of nephrotoxicity.

In this study, treatment with colistin was associated with a lower cure rate in critically ill patients. Critically ill patients often have severe infections associated with *A. baumannii* or highly resistant pathogens with high minimal inhibitory concentrations and variable pharmacokinetics; therefore, they require aggressive treatment. In a retrospective study of patients with pneumonia, conducted in Taiwan, 72% of patients had documented microbiological eradication, 71.6% experienced a clinical cure, and the mortality rate during treatment was 16.4% (Zheng et al., 2020). Compared with patients who received an adequate intravenous colistin dose, those who received an inadequate dose had a significantly longer treatment duration and a significantly higher 30-day mortality rate, and colistin dose was an independent predictor of 30-day mortality. In this study, patients on dialysis, the majority of whom were critically ill, had lower cure rates and higher case fatality rates than those not on dialysis. High PK variability has been observed in patients who received renal replacement therapy (RRT) (Nation et al., 2017; Garonzik et al., 2011; Jacobs et al., 2016). Moreover, both free colistin and CMS are removed by RRT and are adsorbed in part by the hemofilter. Th removal of colistin through different RRT modalities ranges between 55%–76% of colistin via intermittent hemodialysis and adsorption through dialysis membrane (Garonzik et al., 2011; Jitmuang et al., 2015) and between 62% and 67.4% of colistin is removed by continuous renal replacement therapy (CRRT) (Karvanen et al., 2013) and slow low efficiency dialysis (SLED) (Karvanen et al., 2013). Optimal antibiotic dosing in patients receiving RRT is necessary but remains challenging (Jitmuang et al., 2015; Karvanen et al., 2013; Boonyasiri et al., 2025; De Pascale et al., 2024). Findings emphasized the need for higher CMS doses when RRT is used and suggested dosing regimens to achieve clinically desirable colistin plasma concentrations while minimizing nephrotoxicity risks. De Pascale et al. (2024) examined high colistin dose in critically ill patients on CRRT and found that colistin levels were above bacterial MIC_90_ but exceeded the safety average steady sate concertation limit. However, authors reported high mortality rate among the studied population. Therefore, it is recommended to perform early and repeated therapeutic drug monitoring to improve colistin dosing accuracy in individual patients on RRT (Boonyasiri et al., 2025; De Pascale et al., 2024). Clinical outcome studies in patients receiving RRT are limited. The reason for the high mortality in patients receiving dialysis requires further investigation to establish whether it is related to subtherapeutic colistin dosing or to other factors.

This study showed variability in colistin dosing among public hospitals in Kuwait. The variation might be related to clinicians not being aware of the changes, lack of standard protocols in some hospitals, and perception of colistin dosing recommendations. This study included both critically ill and non-critically ill patients, which is unique and previously unreported. Current evidence about colistin usage and dosing recommendations, including recommendations regarding the loading dose, is based on critically ill patients. In this study, some non-ICU patients were administered loading doses of colistin, suggesting that the clinicians who prescribed the medication applied the published recommendations for critically ill patients. To our knowledge, this is the first report of the use and outcomes of colistin administration to non-critically ill patients. Similarly, variable maintenance doses were reported in this study, with the most frequent dose being 9 MIU per day. Such high maintenance doses were associated with improved outcomes and low mortality but a high incidence of nephrotoxicity.

The reported incidence of AKI in this study is similar to the 26.7%–39.1% that has been reported in previous studies and a meta-analysis (Eljaaly et al., 2021; Oliota et al., 2019; Wagenlehner et al., 2021). The association between colistin therapy and a high risk of nephrotoxicity has been reported previously, with patients receiving colistin having approximately double the odds of developing nephrotoxicity compared with that of receiving other antimicrobial therapies (Wagenlehner et al., 2021). High colistin doses were found to be associated with an increased risk of AKI, but the association with the loading dose was unclear. This study showed that a high maintenance dose of colistin (9 MIU per day), but not the loading dose, was associated with a high risk of AKI. These results are consistent with Wagenlehner et al (Wagenlehner et al., 2021) who showed that higher maintenance doses were associated with increased odds of nephrotoxicity compared to lower doses. In this study, older age was also associated with the development of AKI; however, other factors were not explored. In previous a study, older age and concomitant use of diuretics, vasopressors, and glycopeptide antibiotics have been found to be associated with an increased risk of colistin-associated nephrotoxicity (Wagenlehner et al., 2021).

Evidence of colistin neurotoxicity is limited in case reports (Nigam et al., 2015; Sodhi et al., 2014; Wadia and Tran, 2014) and studies (Simon et al., 2023; Kaye et al., 2023) because it is difficult to detect, especially in critically ill patients who are often unconscious. In this study, the reported incidence of neurotoxicity was similar to the 3%–8.3% incidence reported in previous studies (Wagenlehner et al., 2021; Simon et al., 2023; Kaye et al., 2023).

The results of the unadjusted logistic regression analysis showed that the concomitant use of other antibiotics (such as carbapenem, piperacillin/tazobactam, quinolone, or tigecycline) was not associated with improved cure rates, consistent with the findings of two randomised controlled trials (Kaye et al., 2023; Paul et al., 2018) that compared colistin combination therapy with carbapenem with colistin monotherapy for the treatment of pneumonia or bloodstream infections with extensively drug-resistant (XDR) gram-negative pathogens, such as XDR *A. baumannii*, XDR *P. aeruginosa*, and carbapenem-resistant Enterobacterales (Kaye et al., 2023; Paul et al., 2018).

### 4.1 Strengths and limitations

To our knowledge, this is the first study to describe the pattern of colistin therapy use in Kuwait and it provides a basis for comparative studies in the Middle East and elsewhere. Strengths of this study include the exclusion of patients who were neutropenic and those with sterile culture or a culture with non-XDR gram-negative pathogens to minimize bias and confounding factors. This study thus reflects the true colistin-related clinical cure rates in routine clinical practice.

This study has some limitations including the small sample size. Further prospective studies are required to confirm our findings. In this study, most patients did not have a repeat culture after completing colistin therapy; therefore, evaluation of the microbiological cure rate was not possible. Other limitations include the lack of information about severity-of-illness scores, prior antibiotic use in the past 6 months, C-reactive protein as inflammatory biomarker, and the lack of serum colistin concentration data because serum colistin measurement is currently not performed in Kuwait.

## 5 Conclusion

Administration of a colistin loading dose was not associated with clinical cure rate, incidence of nephrotoxicity, or mortality. Administration of a high maintenance dose of colistin was associated with high cure rates, low case fatality rate, but a high incidence of nephrotoxicity. Patients on dialysis had low cure and high case fatality rates. Further research is required to determine the underlying factors and improve treatment success and survival in patients on dialysis. The study findings demonstrate associations rather than causal relationships and prospective studies are needed to confirm the observed dose-outcome relationship. These findings provide baseline data on colistin prescription patterns in Kuwait, which can be used to optimise the use of colistin therapy.

## Data Availability

The raw data supporting the conclusions of this article will be made available by the authors, without undue reservation.
