# Supplementary material for: Colistin use and outcomes in Kuwait public hospitals
Source: Front Drug Saf Regul. 2026 May 8;6:1808461. doi: 10.3389/fdsfr.2026.1808461 (PMC13194472; doi:10.3389/fdsfr.2026.1808461)
Supplement: Supplementary file 1 [file Supplementaryfile1.docx]

**Supplementary material:**

**Table S1: The detected pathogen in ICU versus non-ICU patients.**

|  | **ICU patients (n=117)** | | **Non-ICU patients (n=88)** | | **Total cohort**  **(n=205)** | | **P value *** |
| --- | --- | --- | --- | --- | --- | --- | --- |
| *Acinetobacter baumannii* | 87 | (74.4) | 36 | (40.9) | 123 | (60.0) | <0.0001 |
| *Pseudomonas aeruginosa* | 24 | (20.5) | 38 | (43.2) | 62 | (30.2) | <0.0001 |
| *Klebsiella pneumoniae* | 18 | (15.4) | 26 | (29.5) | 44 | (21.5) | 0.015 |
| *Escherichia coli* | 6 | (5.1) | 6 | (6.8) | 12 | (5.9) | 0.610 |
| *Enterobacter cloacae* | 2 | (1.7) | 1 | (1.1) | 3 | (1.5) | 0.735 |
| *Stenotrophomonas maltophilia* | 1 | (0.9) | 0 | (0.0) | 1 | (0.5) | 0.385 |

Results are presented as n (%). ICU: intensive care unit.

* Chi-square test.

**Table S2: Univariate logistic regression of clinical outcomes.**

|  | **Clinical cure** | | | **AKI** | | | **Death** | | |
| --- | --- | --- | --- | --- | --- | --- | --- | --- | --- |
|  | **OR** | **95% CI** | **P value** | **OR** | **95% CI** | **P value** | **OR** | **95% CI** | **P value** |
| No loading dose | Reference | | | | | | | | |
| Receiving an LD | 0.53 | [0.29, 0.98] | 0.04 | 1.16 | [0.60, 2.24] | 0.66 | 1.85 | [0.95, 3.57] | 0.07 |
| Daily MD < 9 MIU | Reference | | | | | | | | |
| Daily MD ≥ 9 MIU | 2.20 | [1.05, 4.62] | 0.04 | 2.34 | [1.19, 4.60] | 0.01 | 0.36 | [0.15, 0.85] | 0.02 |
| Duration of therapy (days) | 1.06 | [1.001, 1.12] | 0.04 | 1.04 | [0.99, 1.09] | 0.11 | 0.90 | [0.84, 0.97] | 0.00 |
| ICU patients | Reference | | | | | | | | |
| Non-ICU patients | 3.87 | [1.93, 7.76] | 0.00 | 1.09 | [0.57, 2.08] | 0.79 | 0.20 | [0.09, 0.46] | 0.00 |
| Age (year) | 0.96 | [0.95, 0.98] | 0.00 | 1.01 | [1.00, 1.03] | 0.14 | 1.04 | [1.01, 1.06] | 0.00 |
| Male gender | Reference | | | | | | | | |
| Female gender | 0.52 | [0.29, 0.96] | 0.04 | 1.03 | [0.54, 1.96] | 0.93 | 1.35 | [0.70, 2.58] | 0.37 |
| Not on dialysis | Reference | | | | | | | | |
| On dialysis | 0.33 | [0.16, 0.69] | 0.00 | 0.70 | [0.28, 1.71] | 0.43 | 4.15 | [1.93, 8.90] | 0.00 |
| Baseline creatinine clearance | 1.00 | [0.99,1.01] | 0.07 | 1.00 | [1.0006,1.01] | 0.04 | 0.99 | [0.99,1.00001] | 0.06 |
| With concomitant antibiotic | Reference | | | | | | | | |
| Monotherapy | 0.88 | [0.48, 1.63] | 0.68 | 1.25 | [0.66, 2.40] | 0.49 | 1.05 | [0.54, 2.04] | 0.88 |
| Site of infection: Blood | 0.50 | [0.19,1.27] | 0.14 |  |  |  |  |  |  |
| Chest | 0.90 | [0.32,2.51] | 0.84 | 0.91 | [0.34,2.44] | 0.85 | 1.19 | [0.44,3.25] | 0.73 |
| Skin and soft tissue | 3.43 | [0.64,18.26] | 0.15 | 0.71 | [0.24,2.07] | 0.53 | 1.20 | [0.41,3.48] | 0.74 |
| Urine | 2.16 | [0.66,7.10] | 0.21 | 0.88 | [0.22,3.53] | 0.85 | 0.35 | [0.06,1.89] | 0.22 |
| Multiple site infection | 0.50 | [0.19,1.27] | 0.14 | 1.00 | [0.34,2.90] | 0.99 | 0.55 | [0.16,1.87] | 0.34 |
| *Acinetobacter baumannii (no)* | Reference | | | | | | | | |
| *Acinetobacter baumannii (yes)* | 0.60 | [0.32, 1.14] | 0.12 | 1.44 | [0.74, 2.81] | 0.29 | 1.10 | [0.56, 2.14] | 0.79 |
| *Pseudomonas aeruginosa (no)* | Reference | | | | | | | | |
| *Pseudomonas aeruginosa (yes)* | 1.44 | [0.73, 2.84] | 0.29 | 0.84 | [0.41, 1.70] | 0.62 | 0.85 | [0.41, 1.75] | 0.66 |
| *Klebsiella pneumonia (no)* | Reference | | | | | | | | |
| *Klebsiella pneumonia (yes)* | 1.80 | [0.81, 4.03] | 0.15 | 1.33 | [0.63, 2.80] | 0.46 | 0.83 | [0.37, 1.89] | 0.66 |

AKI: acute kidney injury, CI: confidence interval, ICU: intensive care unit, LD: loading dose, OR: odds ratio, MD: maintenance dose.

**Table S3: Detailed multivariable logistic regression of clinical outcomes.**

|  | **Clinical cure** | | | **AKI** | | | **Death** | | |
| --- | --- | --- | --- | --- | --- | --- | --- | --- | --- |
|  | **OR** | **95% CI** | **P value** | **OR** | **95% CI** | **P value** | **OR** | **95% CI** | **P value** |
| No loading dose | Reference | | | | | | | | |
| Receiving an LD | 0.67 | [0.29,1.57] | 0.36 | 0.96 | [0.41,2.24] | 0.93 | 1.96 | [0.75,5.13] | 0.17 |
| Daily MD < 9 MIU | Reference | | | | | | | | |
| Daily MD ≥ 9 MIU | 2.77 | [1.04,7.37] | 0.04 | 2.60 | [1.08,6.23] | 0.03 | 0.29 | [0.09,0.95] | 0.04 |
| Duration of therapy (days) | 1.07 | [1.00,1.14] | 0.05 | 1.06 | [0.99,1.12] | 0.05 | 0.87 | [0.80,0.95] | 0.002 |
| ICU patients | Reference | | | | | | | | |
| Non-ICU patients | 3.04 | [1.23,7.52] | 0.02 | 1.21 | [0.51,2.89] | 0.66 | 0.16 | [0.05,0.49] | 0.00 |
| Age (year) | 0.96 | [0.94,0.99] | 0.01 | 1.04 | [1.02,1.07] | 0.00 | 1.04 | [1.01,1.08] | 0.01 |
| Male gender | Reference | | | | | | | | |
| Female gender | 0.64 | [0.30,1.37] | 0.25 | 0.80 | [0.38,1.67] | 0.55 | 1.12 | [0.48,2.62] | 0.79 |
| Not on dialysis | Reference | | | | | | | | |
| On dialysis | 0.36 | [0.13,0.98] | 0.05 | 0.93 | [0.30,2.83] | 0.89 | 6.78 | [2.21,20.75] | 0.00 |
| Baseline creatinine clearance | 1.00 | [0.99,1.00] | 0.23 | 1.01 | [1.00,1.01] | 0.02 | 1.00 | [1.00,1.01] | 0.10 |
| Site of infection: Blood | Reference | | | | | | | | |
| Chest | 0.84 | [0.27,2.64] | 0.76 | 0.76 | [0.25,2.33] | 0.63 | 0.52 | [0.14,1.97] | 0.34 |
| Skin and soft tissue | 1.07 | [0.32,3.60] | 0.91 | 0.64 | [0.19,2.13] | 0.47 | 0.97 | [0.25,3.73] | 0.96 |
| Urine | 3.09 | [0.46,20.82] | 0.25 | 1.38 | [0.27,6.99] | 0.70 | 0.37 | [0.05,2.82] | 0.34 |
| Multiple site infection | 2.33 | [0.52,10.48] | 0.27 | 1.07 | [0.28,4.09] | 0.92 | 0.71 | [0.13,3.86] | 0.69 |
| With concomitant antibiotic | Reference | | | | | | | | |
| Monotherapy | 0.75 | [0.34,1.65] | 0.47 | 1.67 | [0.75,3.70] | 0.21 | 1.13 | [0.46,2.75] | 0.79 |
| *Acinetobacter baumannii (no)* | Reference | | | | | | | | |
| *Acinetobacter baumannii (yes)* | 1.19 | [0.44,3.18] | 0.73 | 1.47 | [0.53,4.12] | 0.46 | 0.72 | [0.24,2.15] | 0.56 |
| *Pseudomonas aeruginosa (no)* | Reference | | | | | | | | |
| *Pseudomonas aeruginosa (yes)* | 1.24 | [0.48,3.20] | 0.66 | 0.76 | [0.30,1.97] | 0.57 | 1.28 | [0.44,3.75] | 0.65 |
| *Klebsiella pneumonia (no)* | Reference | | | | | | | | |
| *Klebsiella pneumonia (yes)* | 1.43 | [0.52,3.92] | 0.49 | 1.55 | [0.62,3.87] | 0.35 | 0.84 | [0.28,2.50] | 0.75 |

AKI: acute kidney injury, CI: confidence interval, ICU: intensive care unit, LD: loading dose, OR: odds ratio, MD: maintenance dose.

Figure S1: Correlation between dialysis and daily maintenance dose of colistin (correlation coefficient = -0.35, P < 0.0001)
